# The characteristics and experience of community food program users in arctic Canada: a case study from Iqaluit, Nunavut

**DOI:** 10.1186/1471-2458-12-464

**Published:** 2012-06-21

**Authors:** James Ford, Marie-Pierre Lardeau, Will Vanderbilt

**Affiliations:** 1 Department of Geography, McGill University, Montreal, Canada

## Abstract

**Background:**

Community food programs (CFPs), including soup kitchens and food banks, are a recent development in larger settlements in the Canadian Arctic. Our understanding of utilization of these programs is limited as food systems research has not studied the marginalised and transient populations using CFPs, constraining service planning for some of the most vulnerable community members. This paper reports on a baseline study conducted with users of CFPs in Iqaluit, Nunavut, to identify and characterize utilization and document their food security experience.

**Methods:**

Open ended interviews and a fixed-choice survey on a census (n = 94) were conducted with of users of the food bank, soup kitchen, and friendship centre over a 1 month period, along with key informant interviews.

**Results:**

Users of CFPs are more likely to be Inuit, be unemployed, and have not completed high school compared to the general Iqaluit population, while also reporting high dependence on social assistance, low household income, and an absence of hunters in the household. The majority report using CFPs for over a year and on a regular basis.

**Conclusions:**

The inability of users to obtain sufficient food must be understood in the context of socio-economic transformations that have affected Inuit society over the last half century as former semi-nomadic hunting groups were resettled into permanent settlements. The resulting livelihood changes profoundly affected how food is produced, processed, distributed, and consumed, and the socio-cultural relationships surrounding such activities. Consequences have included the rising importance of material resources for food access, the weakening of social safety mechanisms through which more vulnerable community members would have traditionally been supported, and acculturative stress. Addressing these broader challenges is essential for food policy, yet CFPs also have an essential role in providing for those who would otherwise have limited food access.

## Background

Food insecurity is a chronic problem affecting Inuit settlements in Canada. In Nunavut Territory, for instance, 56% of the Inuit population is estimated to be food-insecure [1], with community studies indicating prevalence ranging from 50 to 80% [2-5]. This significantly exceeds the Canadian average and includes high prevalence among children [6]. Food insecurity is reflective of, and in turn contributes to, the poor health status of Inuit, and has been identified as one of the major policy challenges facing northern governments [7-15].

The last two decades have witnessed a proliferation of research examining Inuit food systems, proving insight on food (in)security. Inuit food systems combine interdependence on traditional components based on subsistence hunting and fishing activities – which remain a major source of food [16] – and market based or store foods. Early food research in Arctic Canada focused on sharing in contemporary Inuit society, examining customary rules governing how traditional foods are produced, processed, distributed, prepared and consumed [17-20]. More recently, this work has examined how traditional food systems are changing and what this means for food security at a community level [21-27]. In the early 1990s research also began to examine contaminants in traditional foods and associated health implications, as it became clear that Arctic regions were accumulating contaminants emitted in the south; this remains an important focus of study [28-30]. Concern over contaminants, along with broader interest in dietary change, in turn provided impetus for research documenting dietary consumption patterns, examining change in dietary habits over time, and assessing the adequacy of nutrient intakes [5,6,31-44]. Some of these studies have utilized modified versions of the Radimer/Cornell and USDA household food questionnaire to quantify prevalence of food insecurity [1-5].

The literature develops an understanding of the magnitude of the food security challenge facing Inuit communities and the roles played by various factors. A preference for working in small, remote, and more traditional communities (population <1500) is discernible along with a focus on the traditional food component of the food system. Vulnerable groups including children and females have been the focus of a few recent studies [4,6,45]. The experience and determinants of food insecurity in the larger regional Inuit centers (RICs) (e.g. Iqaluit, Inuvik), however, have been largely unexamined. These regional centers differ significantly from the smaller communities that have been the focus of research, with their administrative functions, rapidly developing economies, transport links, and rapid in-migration, with livelihoods combining a strong dependence on the waged economy alongside continued importance on subsistence-based harvesting activities [46-48].

Employment opportunities in RICs and lower retail prices for store food relative to what is found in smaller communities which are more remote from larger food distribution routes are likely, on the one hand, to moderate food insecurity compared to smaller communities, where poverty and unemployment are major constraints on the ability to access food. Equally, research has indicated that social networks through which traditional foods are shared between and within households are often weaker in RICs, a function of demographics, predominance of livelihoods based on the waged economy, in-migration and transiency in habitation [47-49]. Moreover, the larger settlements, while increasingly prosperous, have significant pockets of inequality, characterized by high and persistent unemployment, poverty, and house overcrowding [50,51]. For this ‘underclass,’ food insecurity is typically chronic and manifest in an inability to access traditional or store foods [48]. In response, community food programs (CFPs) have been initiated in some RICs, including the development of food banks and soup kitchens. Such support mechanisms are a recent development in the Canadian Arctic and reflect the challenges faced in the context of modernization, acculturation, and population growth.

While there is a well developed literature on CFPs in urban areas of southern Canada, very little is known in a Canadian Arctic context, or indeed the Arctic more generally. Who is using CFPs? How often? Why? Is use changing over time? How are food programs perceived? These are important questions, particularly in-light of stressors such as climate change, the rising cost of living, changing sharing networks, and population growth, all of which have the potential to increase demand for formalized food services. Policy makers and program coordinators have identified this knowledge gap as challenging service planning for this segment of the population, who have historically been neglected in food system initiatives [48]. This gap partly reflects the challenging nature of researching marginalised and transient populations using CFPs who are sometimes homeless and living in shelters or in temporary housing. These individuals are unlikely to be captured in research recruiting study participants randomly based upon housing lists and maps (e.g. Inuit Health Survey) or in qualitative studies using convenience or snowball sampling. Rather, they need to be explicitly targeted as a vulnerable sub-population. This paper is situated within this context, and employs a mixed methods research approach to document and examine utilization and the food security experience of users of CFPs drawing upon a case study from Iqaluit, Nunavut. In developing a baseline understanding of utilization and determining factors, we close by examining policy interventions to strengthen current food programming targeted at vulnerable community members.

## Methods

### Case study: Iqaluit, Nunavut

**Figure 1:**
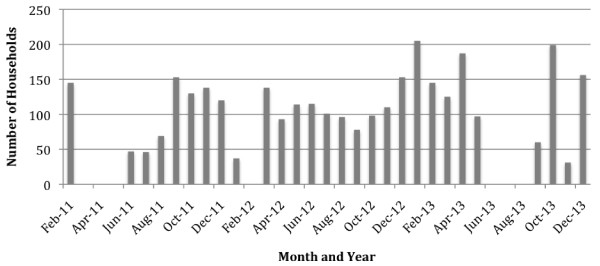
**Number of household units visiting the food bank per month from 2007 to 2009.** Note: Household unit refers to any type of family: single individual, homeless, family living on their own. In 2007, the food bank operated over 7 months and had 18 delivery days. In 2008, the food bank operated over 12 months and had 28 delivery days. In 2009, the food bank operated over 9 months and had 13 delivery days. In 2007 and 2009, the Food Bank was closed during the summer months.

Iqaluit is the territorial capital of Nunavut, Canada, with population of 6185 (58% Inuit) (Table 1). Located on southern Baffin Island at the head of Frobisher Bay (Figure 1), the community’s economy consists primarily of waged employment and many Inuit and non-Inuit are attracted to the area for jobs [47]. Hunting remains a strong part of community life with seal, caribou, walrus, various fish, and beluga whale regularly harvested. Iqaluit is the largest community in Nunavut, and the only location with a hospital, a young offender’s centre, and jails and shelters for both men and women. As a rapidly growing town and magnet for people from other settlements, Iqaluit’s population is more transient than other Nunavut communities. The Inuit population in Iqaluit grew by 17.6% between 2001 and 2006, compared with a 9.2% increase in Nunavut as a whole*.* Iqaluit is one of the few communities in the Canadian Arctic with a number of CFPs providing for those in need, including a food bank with bi-monthly distributions and a soup kitchen that serves daily meals. A drop in centre – the Tukisigiarvik Friendship Centre (“place to find understanding” in Inuktitut) – was established in 2003, with traditional foods are available on a daily basis.

**Table 1:** **Socio-economic demographic data for Iqaluit, based on the 2006 Census**^**1**^

**Indicators**	**Study Participants n (%)**	**Iqaluit, Nunavut n(%)**
**Population (n)**	**94**	**6185**
-Male	53 (56)	3175 (51)
-Female	41 (44)	3010 (49)
Reporting Inuit identity	91 (97)	3540 (58)
Age Group		
-18-24 yrs old	13 (14)	530 (9)
-25-34 yrs old	23 (25)	1215 (20)
-35-44 yrs old	26 (28)	1075 (17)
-45-54 yrs old	23 (25)	780 (13)
-55-64 yrs old	7 (7)	400 (6)
over 65 yrs old	2 (2)	135 (2)
Unemployment rate population over 15 yrs old	67 (72)	275 (8)
Source of income (all family types)^2^		
-*Employment income*	26%	*Data not available*
-*Government transfer payments*	61%	*Data not available*
-*Other income sources*	13%	*Data not available*
Education (for population 15 yrs and over)		
-No certificate, diploma or degree	82 (87)	1615 (36)
-High school certificate or equivalent	3 (3)	775 (17)
-Higher education (trades, apprenticeship, college, university)	8 (9)	2140 (47)

^1^ Nunavut Bureau of Statistics. *Census Data 2006*. http://www.eia.gov.nu.ca/stats/census.html.

Accessed 12 September 2010.

^2^ Statistics Canada. *Community Profiles 2006*. http://www12.statcan.gc.ca/census-recensement/2006. Accessed 12 September 2010.

The challenges facing Iqaluit residents are characteristic of those facing RICs in the Canadian north more broadly. While increasingly prosperous as the capital of one of Canada’s fastest growing regions, inequality in income, employment opportunities, and health outcomes are pronounced [47,49]. In particular, for those without a formal education, who suffer from mental health or addiction-related problems, or have a criminal record, finding a stable job is difficult. Hidden homelessness or house insecurity is also an increasing problem, characterized by an inability of individuals or families to find stable housing [49,51].

### Data collection

A community based participatory research (CBPR) approach guided the study, with research questions identified during consultation with territorial level policy makers, local leaders, community members, and northern science bodies. This was followed with a one week photovoice workshop with 8 regular users of the food bank to assist with project development and identify research needs and questions [48]. This work informed the creation of a mixed-methods approach to data collection involving a fixed-choice survey and open ended questions with the aim of documenting and examining CFP utilization and food security experience. Mixed methods enabled the collection of standardized data and quantitative analysis while also allowing users to describe in their own words their experiences. The research team – consisting of university-based researchers, community health professionals, and two local research assistants who were users of food programs – evaluated the results with participants. The research followed ethical norms for working with communities in northern Canada, including obtaining university research ethics board consent from McGill University (REB#: 65–0710), a research license from the Nunavut Research Institute (#0104810 NA), eliciting informed consent from all study participants, and ensuring confidentiality of participants.

A census of clientele using CFPs during May 2010 (n = 94) was conducted. This involved the research team visiting the food programs during hours of operation, at which time users were randomly asked to participate. Sampling continued over a 4 week period until theoretical saturation was reached, at which no new users were identified. Each participant was first asked a series of fixed-choice close-ended survey questions covering socio-demographic-livelihood characteristics, food access, and frequency of use of CFPs (Table 2). A locally adapted version of the U.S. Department of Agriculture Food Security Module (FFSM) was then administered. Due to the fact that the target population consisted of regular users of CFPs, it was assumed that they already experienced food insecurity and the survey therefore used only 4 locally adapted questions of the standard 6 item subset of the 12 month FFSM, which are also part of the core domains of the food insecurity experience shared across cultures [52]. These questions focus on the experience of not having enough food in the household, reducing food portions and switching to cheaper foods. Questions on coping strategies were adapted from the coping strategies index developed by Maxwell et al. [53]. Following the survey, open-ended questions were asked in order to examine and document, in the user’s own words, perceptions of the services offered, why they were using the food programs, and the nature of food insecurity experienced.

**Table 2:** Key areas explored in the surveys and open-ended questions

**Survey – Key areas**	**Open-ended questions**
- Socio-demographic information: *birth town, sex, age group, education level, number of people in household, income level, etc.*	- Perception of services: *Are they helping your situation? How so? How do you feel when you use the services?*
- Access to country foods: *hunter in the household, access to sharing, main source of country foods, etc.*	- Challenges to be food secure: *What are the main obstacles to be food secure?*
- Food security and coping strategies: *not enough food in the past 12 months, reducing portions for oneself or others in the household, skipping meals, selling belongings, etc.*	- What are the most difficult times during the month, during the year? What makes those times more difficult to have enough food?
- Frequency of use of services: *how often, since when?*	- How can the services be improved?

Participants received a $40 CAN gift card for the local supermarket for their time and all questions were asked in the language of choice (English or Inuktitut). Recruitment and interviewing took place during hours of operation of the respective programs and in a private setting to ensure confidentiality. The questions were pre-tested and evaluated by the team to ensure appropriateness and effectiveness. Additional data was obtained from handwritten records on utilization kept by the food bank (for 2007–2009), soup kitchen (for 2005–2009), and Tukisigiarvik (for 2003–2009), which were entered into excel for analysis. Key informant interviews were also conducted with the territorial nutritionist, personnel at the CFPs, and the Quajigiartiit Health Research Network, the Nunavut Research Institute and the Inuit Institute for Research and Planning to obtain multiple perspectives on utilization, and complimented by participant observation in which researchers volunteered at the various CFPs.

### Analysis

Data were analyzed in SPSS 15.0. Basic descriptive statistics were used to describe the sample population, responses to each question, and to ascertain the distribution of responses by age, sex, occupation, and hunting behaviour. Chi-squared (*x*^2^) analysis and Fischer exact tests were performed to test for significant differences between participant characteristics and response to questions, and to compare the sample population with that of Iqaluit (where community-level data was available). Significance was set at p < 0.05. Open-ended questions were used to explore participant’s experiences and perceptions of CFPs in Iqaluit. Coding was used to sort qualitative answers in content related categories by using non-automated frequency counts and through latent content analysis.

## Results

Ninety four interviews were conducted. Socio-demographic characteristics of the sample population are provided in Table 1.

### Community food programs in Iqaluit are widely used and valued

**Figure 2:**
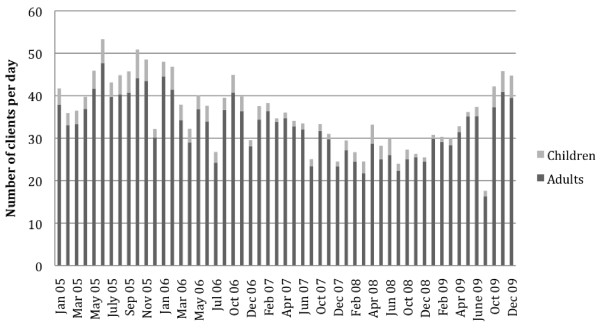
**Average number of adults and children attending the soup kitchen for lunch on a monthly basis.** Peak attendance at the soup occurred in 2005 (43 clients per day), when the soup kitchen was serving lunch and supper seven days a week. In recent years the soup kitchen’s operation reduced to serving one meal per day (lunch), five days a week. In 2009, it served meals to an average of 36 people per day.

In 2008–2009, the food bank distributed food to 365 households (18.1% of total number of households in Iqaluit based on 2006 census). Peaks in attendance occur in early winter (November, December), while fewer clients used their services in the fall and summer (Figure 1). The soup kitchen had an average of 275 days of operation per year between 2005 and 2009, and serves an average of 9984 meals annually, primarily to adults (Figure 2). The soup kitchen had its peak attendance in 2005, receiving an average of 43 people per operation day during its first year of operation when it was open seven days per week for lunch and dinner, and every month of the year. In recent years the soup kitchen’s operation has been curtailed with a reduction of service from two meals per day seven days a week, to one meal per day five days a week. In 2009, it served meals to an average of 36 people per day. Tukisigiarvik received 3828 drop-ins in 2008–2009, an increase from the 3690 drop-ins that were made in 2004–2005. These drop-ins include stopping for counselling, sharing traditional foods, doing laundry and participating in cultural activities.

"*"When I come [to the soup kitchen], it reduces the number of people to feed in my house" (female, 35–44 yrs old, full time worker)*"

"*"We don’t have a choice, we don’t have money to buy food" (male, 45–54 yrs old, unemployed)*"

"*"The food bank is very helpful, especially having 2 small kids who don’t understand there is no food"(female, 45–54 yrs old, unemployed)*"

"*"We can’t find people to help. If it weren’t for these services, we would go hungry" (female, 45–54 yrs old, unemployed)*"

CFPs were highly valued among users, with 82% reporting that they regularly help alleviate hunger, while the absence of other options for accessing food during times of need was widely noted. Participants explained that these programs provide more than a source of food, increasing a sense of well-being by decreasing anxiety about not being able to afford food and reducing feelings of helplessness:

"*"I feel really bad when children are hungry, and I like knowing that these organizations can help them" (female, 18–24 yrs old, unemployed)*"

"*"Makes it easier, because I have no money or work" (female,35-44 yrs old, unemployed)*"

"*"When I get very depressed from hunger, they lift me up" (female, 45–54 yrs old, part time worker)*"

"*"It would be really stressful if [the food programs] didn’t exist" (male, 25–34 yrs old, unemployed)*"

"*"There would be a lot more social problems without these organisations" (male, 25–34 yrs old, unemployed)*"

### Repeat use is common

The food bank was used at least once a month for 79% of respondents, the soup kitchen was used two times or more per week for 81%, and Tukisigiarvik was used at least two times per week for 70% of the respondents. Sixty two percent reported using all three services, with the majority using the CFPs for one year or longer (65% for the food bank, 59% for the soup kitchen and Tukisigiarvik). CFPs are typically the main source of food for these users, and often represent the most reliable source they can access. There was no association between gender and frequency of use of CFP’s, although men were more likely to report going to Tukisigiarvik then women (p = 0.05). Finally, there was no association between employment status (employment defined as a wage earning activity and including part time work), nor place of birth and frequency of use of CFP’s. However, a small sample size, particularly for those employed and not using CFP’s, is likely to have reduced statistical power to detect an association between employment and CFP use. A larger sample size would be required to detect an association.

### Users are primarily Inuit, born in Iqaluit, unemployed and did not complete high school

Comparing the prolife of CFP users with socio-economic indicators of Iqaluit obtained from the 2006 census indicates that users are significantly more likely to be Inuit (p < 0.01 Fisher’s Exact test), with 97% self identifying as Inuit compared to 58% for the community as a whole. Users were also more likely to be unemployed (p < 0.01 Chi-squared test), with 72% reporting to be unemployed at the time of the survey: eight times greater than the community as a whole. Twelve percent reported working full time, 7% worked part time, 4% were full-time hunters or fishers and 2% were involved in traditional craft production (i.e. carving). Significantly fewer users reported completing high school (13%) (p < 0.01 Chi-squared test) compared to the general population, where 66% completed high school. Twelve percent reported having some post secondary education, primarily involving learning a trade. No statistical relationships were documented between users and the general population for gender, where 56% of respondents were male and 46% female, or age.

The majority of users (76%) were born in Iqaluit and of those born elsewhere, over three quarters were from other Nunavut communities and two thirds had lived for more than five years in Iqaluit. Social assistance was the main source of income for 61% of respondents, whereas employment was the main source of income for 26% of participants, with another 13% reporting other benefits or non waged work, such as carving, as their main source of income (Table 1). While the majority of employed participants said that the main source of income in their household was from employment, some said that although they were employed, the main source of income in their household was from income support. In some cases, participants lived in households where they were the only employed member of the household and their salary could not support all members in the household. In such cases, they considered that the main source of income at the household level was from income support.

When asked if income was enough for their needs, 57% of respondents answered rarely or never. Lack of adequate shelter was a concern for many users, with 16% of respondents either homeless or living in shelters at the time of the interview. Others reported living alone (23%), in households with 2 people (16%), 3–4 people (31%), 5 to 8 people (21%), and 9 or more (9%). Of those participants (46%) who said that there where times during the year when they had more people in their house, 30% said that the reason for this was because they had to help out family members and friends who had nowhere else to stay. Lack of shelter was identified as a major constrant to accessing food. As one partcipant said,*"It is hard to have food in the house when there is no house." (male, 25–34 yrs old, homeless)*

### The majority of users live in households without hunters

Three quarters of respondents reported living in a household without a hunter, which was described as making it difficult to obtain traditional food on a regular and predictable basis. While most (72%) reported that they could access traditional foods through sharing, this is dependent on the hunting success of family members and friends and on their willingness to share. Close to one third (28%) of the respondents said that they did not have anyone who could share country food with them, and there was no difference between men or women. Tukisigiarvik provides an important source of traditional foods herein and represented the main source of traditional foods for 33% of respondents. Equally, living in a household with a hunter did not protect against running out of food in the house and being unable to acquire more, as there was no statistically significant difference in the number of households with or without a hunter reporting running out of food in the past year. Also, there was no statistically different level of use of any of the three community food programs between households with a hunter and households without a hunter.

### Users are regularly not able to access food and CFP use is one of a number of coping mechanisms

Running out of food was a major concern for the majority of the respondents, with 89% reporting that in the last year there had been times where they had no food in the household. Along with relying on CFPs, coping strategies documented to manage lack of food access included switching to cheaper foods that were often less preferable (87%), reducing portion sizes (72%), reducing portion sizes of other members of the household (60%), sending people to eat elsewhere (53%) and selling belongings to get money for food (49%). Due to the sensitive nature of this question, we did not ask for a description of those items, yet some study participants did specify having to sell hunting equipment to access money. We did not detect any statistically significant differences between males or females, presence or absence of a hunter in the household or being born in Iqaluit in the responses on coping strategies or food security. Reasons given to explain why there had been times without food in the household included: lack of money (36%), unemployment (27%), having to help others (12%), addictions (8%) and food being too expensive (6%).

### Early winter is the most difficult time to access food

Over half of the respondents (54%) said that early winter (November, December) was the most difficult time of the year to access food, with fewer reporting fall or spring (9%) or the summer (7%), to be difficult times. One fifth (21%) of respondents said that they saw no difference in difficulties accessing food between seasons. Utilization data indicates that these are generally the times of greatest usage of CFPs, although the records are temporally limited. One fifth of respondents reported that there were no differences between seasons when accessing food. Reasons given to explain why certain times were more difficult than others during the year were because there was less hunting in the community (31%), bad weather (14%), because expenses go up during that time (15%) and due to unemployment or lack or stable employment (10%). Also, 17% of respondents mentioned that the most difficult time of the year was when services providing food where closed. Services sometimes close during bad weather events, holidays, the summer or when their own staff or volunteers are in insufficient numbers to operate the facilities. When asked about difficult times of the month, the majority (45%) said it was when they had insufficient money in the household, mostly due to income support being too low, and while they were between cheques (36%).

"*"In the winter, there are less country foods. Foods at the store are more expensive, there are more things to pay for"*"

"*"Between income support cheques, I am broke"*"

### Limited access to financial means reported as the main challenge to achieving food security

Finally, when asked about the main challenge to achieving a sense of food security at the household level, 35% of participants answered unemployment. Closely following unemployment was income support being too low or not having enough money (26%) and the need to support other members of the family or household crowding (14%). The high cost of food was the main difficulty for 12% of respondents, dealing with addictions was the main challenge for 8% of participants and 6% of answers had various other reasons.

## Discussion

To our knowledge, this is the first study to document and examine utilization of CFPs in the Canadian Arctic, and the Arctic more generally. The data provide a snapshot of utilization at a specific point in time (May 2010), and while we interviewed all users over a 4-week period, we recognize that we will have missed those who use the programs at other times of the year. The study describes users of CFPs who typically have a low level of formal education, are unemployed, rely on social assistance, and have a low household income. This is not surprising, with utilization profiles similar to those in southern urban centres [54,55]. Contrary to our expectation that new arrivals to Iqaluit would be overrepresented in utilization of CFPs reflecting the breadth and quality of their social ties, the majority of users were born in Iqaluit, and of those from elsewhere, the majority had been living in Iqaluit for more than five years. Lack of shelter is a challenge facing many CFP users, especially ‘hidden homelessness’ characterized by a lack of a secure and permanent dwelling, involving individuals and families moving from one temporary housing situation to another [51,56]. These situations are typical for users of CFPs, and compound challenges of finding a job, getting an education, recovering from previous trauma, and achieving food security. Housing is a broader problem in Nunavut, where overcrowding in substandard houses is widespread [47,51,57]. While addictive behaviour was reported by 9% of participants as a reason why they had difficulties obtaining food, this likely reflects the nature of the research approach (i.e. formal survey/interview), with key informants and some users noting that addiction is major problem facing those using CFPs. We did not detect an association between employment status, place of birth, gender, age, presence of hunter in the household and utilization of CFPs. This is likely due to both low sample size, as well as low variation in the data.

The role of low income, limited educational achievement, unemployment, hidden homelessness, and addictive behaviour represent proximate causes of food insecurity and CFP use, and form part of what Coates et al. [58] describe as a ‘cluster of problems’ affecting food systems in multiple geographic and cultural contexts. These challenges are particularly acute in Arctic Canada where the cost of living and reliance on a limited number of economic sectors is high [59]. Food in Nunavut, for example, on average costs twice as much in southern urban centres with household income significantly lower [45,59,60]. Ultimately, these causes can only be understood in the context of sweeping socio-economic transformations that have affected Inuit society over the last half century as former semi-nomadic hunting groups were resettled into permanent settlements beginning in the 1950s and incorporated into a colonial relationship with the Canadian state, detailed descriptions of which are provided elsewhere [12,17,61-63]. Iqaluit was one of the first permanently settled communities in the eastern Arctic, beginning with the building of a US Air Force base in 1942 [47,62,64]. This was accompanied by rapid socio-cultural change with the associated development of the formal education system, relative decline in hunting, expansion of the wage-based economy, and rapid population growth [17,65-69], with implications for how food is produced, processed, distributed, and consumed, and hence food security.

The *nutrition transition* is one consequence of these broader influences, with the rising consumption of store foods at the expense of traditional foods widely documented across Inuit communities [4,33], access to which is determined by monetary resources in contrast to the moral economy of reciprocity and exchange that governed access to traditional food [17,18,70,71]. There was historically little need for formalized food programs, as household sharing networks would ensure food access (except during periods when wildlife resources were scarce). Sharing networks continue to be important for Inuit – differentiating the northern CFP experience from that in the south – and the majority of respondents reported that they could access traditional foods through such networks, which is important given the limited number of participants who reported having hunters in the household. Nevertheless, as documented here and elsewhere, in a contemporary context in which hunting is expensive, harvest success is being affected by climate change, and fewer people are engaging in harvesting activities, increasing demands are being placed on diminishing supply of traditional foods [21,61,72-78]. Sharing outside of the household – the primary means of traditional food access for CFP users – is therefore often unpredictable and infrequent, especially for those with less to offer in terms of reciprocity (e.g. material resources). For the more vulnerable members of the community who do not have access to financial resources or have stable housing, this diminished social safety net leaves few alternatives but to use CFPs. The vulnerability of sharing networks is particularly apparent in enhanced CFP usage in November and December, times of the year when traditional foods are hard to access due to ice freeze-up and uncertain snow conditions on inland trails which limit the ability to hunt [73,79]. At these times, sharing concentrates among those in the immediate household and for elders.

Another consequence of these broader changes has been significant *acculturative stress* among northern populations, linked to the rapid changes in livelihoods and culture, and experience of residential schools [10,12,80-83]. Many of the older respondents were born and raised in small hunting camps, resettled in Iqaluit, spent time at tuberculosis sanatoria in southern Canada in the 1950s and 60s, and now live in a modern community. The significant associated acculturative stress and intergenerational trauma provides the context for many of the social and health challenges facing Inuit communities including Iqaluit [13,74]. Thus the financial constraints faced by many CFP users reflect more than the cost of food and unemployment; they are exacerbated by household financial management skills, problems of addiction, and poor living conditions associated with acculturation and recent development of the monetary economy [63,82,84]. Similarly, problems of addiction need to be situated in the context of past abuse and also changing relationships with the environment. The land is a fundamental component of Inuit culture and central to health and well-being – both through the act of hunting and being on the land, and also the act of sharing and consuming traditional foods – yet for CFP users this link was often lacking: few were able to afford to hunt, instead relying on sharing for traditional food access. This directly affects food availability, but also has broader ramifications for well-being [85].

In-light of the magnitude of food insecurity in the Canadian north, food policy is increasingly recognized as a central component of anti-poverty/community development strategies. Addressing the broader determinants of food insecurity is essential for such policy interventions, yet the pervasiveness and persistence of these causes despite recognition at a policy level is indicative of the challenging nature of intervention required. Moreover, at a community level, such ultimate causes are often beyond the scope of what can be achieved, necessitating broad-scale territorial and federal involvement [14]. In this context, while CFPs do not address root causes, they provide a valuable service for a community undergoing rapid change. While it has been argued in the general scholarship and among some northern commentators that CFPs can create dependency and thereby increase food insecurity in the long-term, users in Iqaluit have few alternative sources of food, and numerous barriers make it difficult for users to obtain waged employment necessary for food access. CFPs also provide much more than food. They offer a safe place to go, and in the case of Tukisigiarvik, provide access to culturally important traditional foods which many otherwise would not have access to. Herein, this work suggests a number of priorities for food policy in Iqaluit:

· *Continuing support for CFPs.* While the food bank and soup kitchen rely on donations, Tukisigiarvik recently lost most of their funding as their grant from the Aboriginal Healing Foundation came to end in 2010. The center represents an intervention developed by Iqaluit’s Inuit population after consultations identified the need for a wellness, counselling and advice center to help Inuit in Iqaluit cope with the health and social issues they face. It draws upon traditional approaches that recognize food insecurity to be representative of broader socio-cultural challenges, and as such provides more than just food for those in need including counselling, cultural activities, advice on life skills, and structure.

· *Promotion of traditional foods at the food bank and soup kitchen*. Many participants expressed their gratitude towards the foods offered at the food bank and soup kitchen. However, many also expressed desire to see traditional foods being incorporated in the menus of the soup kitchen, or being offered at the food bank because of the difficulty they have obtaining them otherwise, and more diversity of foods offered. Currently however, the food bank can not serve traditional foods harvested locally because of food safety regulations.

· *Education on how to make the best of store foods offered at the food bank.* Items offered through the food bank are through local donations, and users often reported not knowing how to make use of the food received. The development of cooking classes, pamphlets with recipes, and workshops that teach users how to get the most nutritional value out of the distributed foods were identified as important, and could be undertaken at little cost *.*

· In addition, a number of broader initiatives are needed to strengthen the traditional food component of the food system. Maintaining access to traditional foods is widely recognized as essential for secure food systems in Inuit communities, providing culturally valued nutritious food [14,59].

· *Enhanced support mechanisms to ensure that CFP users can access hunting equipment.* Many participants reported having to sell hunting equipment to access money to buy food, resulting in short term access to material resources but loss of the means of harvesting in the long term, with associated food security implications. Many also reported having hunting skills but no equipment, or could not go hunting because of the cost associated with hunting. A number of hunter support mechanisms are available in Nunavut, and were reviewed by Ford et al. [73,86]. The challenge for CFP users is that many would not meet the requirements for such support mechanisms, while in a community the size of Iqaluit demand for assistance significantly exceeds the resources available. An alternative intervention to the individual focused grants aimed at full time hunters would be a co-op system to allow community members without equipment to access hunting gear. This would provide access for those who want to harvest part-time, prevent people from feeling the need to sell hunting equipment, and spread limited resources around the community. As one participant said: *"I used to be able to hunt before moving here, but not anymore, because I don’t have gear"* (male *, 25–*34 years old, unemployed.

· *Sharing networks to distribute country foods need to be preserved and facilitated:* Sharing of traditional foods remains important in Iqaluit and for CFP users, although the long-term sustainability of such practices has been questioned in light of socio-economic transformations. Initiatives that facilitate the sharing of traditional foods are needed, with community freezers, reduced cargo cost for shipping of traditional foods between communities, support for the new traditional food market in Iqaluit, subsidies on traditional foods sold at stores, and subsidies to hunters to allow them to go hunting, all offering potential entry points.

## Conclusions

Community food programs (CFPs) provide an important service to chronically food insecure households in Iqaluit, Nunavut. Users are among the most vulnerable community members, and typically have low level of education, are unemployed, have limited access to material resources, and have insecure housing. These challenges reflect a complex socio-cultural-political context reflecting rapid transformation in Inuit livelihoods over the past half century, combined with the high cost of living in the north. The baseline study identifies a number of community level priorities for intervention including continuing support for CFPs, promotion of traditional foods at the food bank and soup kitchen, education of using foods offered through CFPs, alongside broader policies to support the harvesting sector. Future research will examine policy interventions in greater depth, with the territorial and federal government increasingly cognizant of the challenges faced by the most vulnerable of Arctic residents.

## Competing interests

The authors declare that they have no competing interests.

## Authors' contributions

JF and MPL conceived the study and participated in its design and coordination. MPL and WV conducted the field work in Iqaluit. MPL conducted statistical data analysis. JF and MPL wrote the manuscript. All authors read and approved the final manuscript.

## Pre-publication history

The pre-publication history for this paper can be accessed here:

http://www.biomedcentral.com/1471-2458/12/464/prepub
